# Paranasal Sinus Wall Erosion and Expansion in Allergic Fungal Rhinosinusitis: An Image Scoring System

**DOI:** 10.7759/cureus.6395

**Published:** 2019-12-16

**Authors:** Surayie Al-Dousary, Ibrahim Alarifi, Amal Bin Hazza’a, Ibrahim Sumaily

**Affiliations:** 1 Otorhinolaryngology, King Saud University, King Abdulaziz University Hospital, Riyadh, SAU; 2 Otorhinolaryngology, Security Forces Hospital, Riyadh, SAU; 3 Otorhinolaryngology, King Abdulaziz University Hospital, Riyadh, SAU

**Keywords:** allergic fungal rhinosinusitis, fungi, allergy, multidetector computed tomography, paranasal sinus, grading system, bone erosion score

## Abstract

Background

Bone erosions are common in allergic fungal rhinosinusitis (AFRS). This study aimed at developing an image-based grading and scoring system for paranasal sinus (PNS) wall erosion in AFRS.

Methods

A retrospective review of all confirmed AFRS cases based on the Bent and Kuhn criteria was conducted. Preoperative computed tomography (CT) images were studied to detect PNS wall erosion with expansion. Based on our observation, we described a grading system based on the proportion of PNS wall erosion, with 1 if less thanone-third, 2 if between one-third and two-thirds, and 3 if more than two-thirds of the wall is eroded. This method provides a new scoring system ranging from 0 to 72. The inter-observer reliability of this scoring system was tested and the percent of agreement was found to be 90%.

Results

Among 142 AFRS cases, 82 patients (57.7%) had bone erosion. Orbital extension via lamina papyracea erosion occurred in 28.2% and 17.6% of the anterior and posterior ethmoid sinuses respectively, via floor erosion in 8.3% of the frontal sinuses, and via roof erosion in 2.1% of the maxillary sinuses. Intracranial extension caused by the anterior skull base erosion occurred in 19.4%, 10.9%, and 6% of the posterior ethmoid, anterior ethmoid, and frontal sinuses, respectively. The middle and posterior cranial fossa skull base was eroded in 14.4% and 9.2% of the sphenoid sinuses, respectively. Infratemporal extension occurred via erosion of the sphenoid sinus lateral wall in 17.3% of the sphenoid sinuses and via erosion of the maxillary sinus posterior wall in 6.7% of the maxillary sinuses. The mean of bone erosion score was 9.52, and the highest score was 34/72.

Conclusion

The orbit is the most common extra-sinus extension site via the lamina papyracea erosion. We propose a new grading and scoring system to assess disease severity and progress.

## Introduction

Allergic fungal rhinosinusitis (AFRS) is a disease related to fungal colonization and the hypersensitivity of specific immunocompetent individuals [1]. The disease was first observed by McCarthy and Pepys in 1971 [2]. In 1981, AFRS was defined as an entity by Millar et al. after discovering similarities in the histological samples of five patients with chronic fungal sinusitis and allergic bronchopulmonary aspergillosis, and they called it “allergic aspergillosis of the paranasal sinuses” [3]. Years later, the name was changed to allergic fungal sinusitis because other fungal species were identified in histopathological samples [4]. In 1994, Bent and Kuhn first described a set of AFRS diagnostic criteria consisting of type I hypersensitivity confirmed by history, skin tests or serology, presence of nasal polyposis, characteristic computed tomography (CT) signs, and eosinophilic mucus without fungal invasion into the sinus tissue, and positive results on fungal staining of sinus contents removed during surgery. They also mentioned other less common findings, including radiographic evidence of bone erosion, positive results on fungal culture, unilateral predominance of the disease, history of bronchial asthma, Charcot-Leyden crystals, and peripheral eosinophilia, which support the diagnosis but are not necessarily present [1].

AFRS develops slowly in susceptible patients and demonstrates non-specific symptoms of chronic rhinosinusitis (CRS) refractory to medical therapy. Some patients may present late with an orbital or intracranial extension resembling an invasive tumor, which occurs by the process of bone erosion and expansion, although invasion of the fungi into the orbital or intracranial tissue does not occur [5-7]. In the case of AFRS, bone erosion is thought to be caused by pressure atrophy from the accumulation of fungal debris and the subsequent expansion of the paranasal sinus (PNS) walls and by the secondary effects of inflammatory mediators [5,8-11].

Several studies have described the bone erosion in AFRS [5,8,12-14], but none has yet mentioned a grading system of PNS wall erosion based on the proportion of the eroded wall. Thus, this study aimed to develop an image-based grading and scoring system for PNS wall erosion in AFRS patients. Instead of using absolute measures, we suggest a relative grading system, as sinus sizes vary among patients. This grading and scoring system will also facilitate longitudinal observations to describe the follow-up of bone erosions and regeneration rates after AFRS treatment.

## Materials and methods

The study protocol was approved by the Institutional Review Board at King Saud University, Riyadh, Saudi Arabia. The medical charts of all patients with a suspected diagnosis of AFRS who were referred to the Otorhinolaryngology Clinic of King Abdulaziz University Hospital at King Saud University between March 2008 and January 2018 were retrospectively reviewed. Only patients who fulfilled the Bent and Kuhn criteria for a diagnosis of AFRS were considered for enrollment [1]. Patients with recurrence after a previous sinus surgery, diagnosis of a bone disorder, or a history of major facial trauma or fractures were excluded.

Demographic data, including age at diagnosis and sex, were collected for all cases. For each patient, the preoperative noncontrasted thin-cut CT of the PNS was studied to detect PNS involvement, wall erosion with expansion, and extra-sinus extension into the orbit, intracranial region, or infratemporal fossa. We used the Lund-Mackay scoring system for the assessment of PNS opacification. This system is widely used for CT evaluation of patients with chronic rhinosinusitis, where 0 indicates no opacity, 1 indicates partial opacification, and 2 indicates complete opacification of the PNS [15]. Each PNS was evaluated to detect any bony erosion with expansion in the clinically relevant PNS walls. We defined erosion as a loss of continuity of the involved PNS wall in more than one CT cut. Loss of continuity without expansion and opacification of the PNS was not counted because it was not considered to be related to AFRS. These cases are usually caused by congenital dehiscence of the PNS wall, which is consistent with the finding of Nussenbaum et al. that all patients with AFRS-related bone erosion have an expansion of the PNS wall [14].

Based on our observations, we established the following three-point grading system for PNS bone erosion in patients with AFRS:

· Grade 1, less than one-third of the wall is expanded and eroded

· Grade 2, more than one-third but less than two-thirds of the wall is expanded and eroded

· Grade 3, more than two-thirds of the wall is expanded and eroded

This clinically relevant descriptive grading system yielded a new way of scoring bone erosion in a patient with AFRS, which we have named the bone erosion score (BES). This score ranges from 0 to 72, where 0 indicates no erosion and 72 indicates that grade 3 erosion is present in all clinically relevant PNS walls. We considered 12 walls of each side to be clinically relevant PNS walls, as follows:

· Three walls of the frontal sinus, i.e., the floor and the anterior and posterior tables

· Two walls of the maxillary sinus, i.e., the roof and posterior walls

· Two walls of the anterior ethmoid sinus, i.e., the roof and lateral wall

· Two walls of the posterior ethmoid sinus, i.e., the roof and lateral wall

· Three walls of the sphenoid sinus, i.e., the roof and the lateral and posterior walls

Erosion of these walls may lead to an orbital, intracranial, or infratemporal extension, which has important implications during endoscopic sinus surgery. We excluded the anterior wall and floor of the maxillary sinus, the floor of the sphenoid sinus, and the medial walls of all sinuses because they are not considered clinically significant. The CT images were evaluated by two rhinologists. CT images of the PNS from 30 patients were selected to test the inter-observer reliability of the grading and scoring systems.

**Figure 1:**
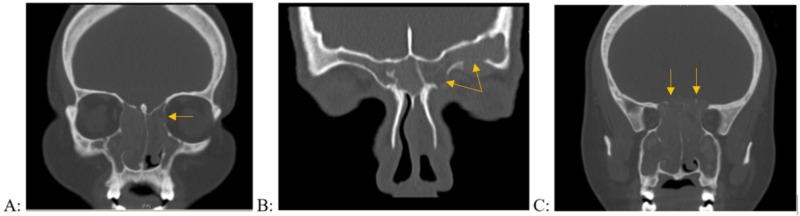
Application of the bone erosion grading system on computed tomography images of the paranasal sinuses in patients with allergic fungal rhinosinusitis (A) Grade 1 erosion of the left lamina papyracea, (B) Grade 2 erosion of the left frontal floor, (C) Grade 3 erosion of the posterior ethmoid roof on the left and right sides

We created a data sheet (Table 1) to grade and score the bone erosion based on the CT findings for each patient. Typical examples of the bone erosion grading system are shown in Figure 1. We determined the incidence rates of orbital wall erosions with orbital extension, skull base erosions with intracranial extension, and infratemporal extension.

**Table 1:** Bone erosion grade of clinically relevant paranasal sinus (PNS) walls. The bone erosion score (BES) is calculated as the sum of all grades, which reaches a maximal value of 72 for a patient.

Paranasal Sinus	Clinically Relevant Wall	Erosion Grade Out of 3	
		Right	Left
Frontal	Floor	
	Anterior		
	Posterior		
Maxillary	Roof	
	Posterior		
Anterior Ethmoid	Roof	
	Lateral		
Posterior Ethmoid	Roof	
	Lateral		
Sphenoid	Roof	
	Posterior		
	Lateral		
Bone Erosion Score (BES)		/72	

At our center, the standard treatment for patients with AFRS is primarily endoscopic sinus surgery, with the aim of opening all the diseased sinuses and cleaning out all the fungal debris. This surgery facilitates the delivery of medication into the sinuses in the postoperative period. All the patients received intranasal steroids and nasal irrigation with normal saline. However, the duration of treatment is variable and depends on the individual case and the preference of the surgeon.

All the study data were entered into a database designed to register the involvement of each sinus wall separately. The results are expressed as the mean and standard deviation (SD) for continuous variables (age and BES) and as number (n) and percentage for categorical variables (patient sex and bone erosion rate). The data were analyzed using Statistical Package for Social Sciences version 23.0 software (IBM Corp., Armonk, NY, USA).

## Results

One hundred and forty-two of 251 patients suspected to have AFRS met our eligibility criteria. Thirty were pediatric patients and 112 were adult patients. The mean patient age was 27.64 years (standard deviation (SD) 10.86), 44.4% were male, and 55.6% were female. Twenty-one frontal sinuses were aplastic, so a total of 1,399 sinuses were evaluated for sinus involvement and the presence and extent of erosion with expansion. The Lund-Mackay score was 0 in 18.9%, 1 in 16.8% and 2 in 64.3% of the examined sinuses. The sinuses most commonly opacified were the posterior and anterior ethmoid sinuses (87.0% and 86.3%, respectively) while the frontal sinus was the least opacified cavity (73.9%).

Eighty-two (57.7%) of the 142 cases had bone erosion with expansion in at least one sinus wall. The mean age of these patients was 23.37 years (SD 8.57). Orbital extension occurred via erosion of the lamina papyracea in 28.2% of the anterior ethmoid sinuses and in 17.6% of the posterior ethmoid sinuses via erosion of the floor in 8.3% of the frontal sinuses and via erosion of the roof in 2.1% of the maxillary sinuses. Erosion of the anterior skull base led to an intracranial extension in 19.4% of the posterior ethmoid sinuses, in 10.9% of the anterior ethmoid sinuses, and in 6% of the frontal sinuses. An intracranial extension also occurred via erosion of the roof in 14.4% and via the posterior wall in 9.2% of the sphenoid sinuses. An infratemporal extension occurred in 17.3% of the sphenoid sinuses via erosion of the lateral wall. An infratemporal extension also occurred in 6.7% of the maxillary sinuses via erosion of the posterior walls (Table 2).

**Table 2:** Incidence of bone erosion in clinically relevant paranasal sinus walls NA, not applicable

Clinically relevant wall	Paranasal sinus				
	Frontal	Maxillary	Anterior ethmoid	Posterior ethmoid	Sphenoid
Roof	NA	2.1%	10.9%	19.4%	14.4%
Floor	8.3%	NA	NA	NA	NA
Anterior	4.9%	NA	NA	NA	NA
Posterior	6%	6.7%	NA	NA	9.2%
Lateral	NA	NA	28.2%	17.6%	17.3%

The mean BES value was 9.15 (SD 7.81). The highest reported score was 34, which was recorded in two patients. Thirty patients were selected for testing the inter-observer reliability of the BES. The inter-observer agreement was found to be 90%.

## Discussion

This study was performed to develop an image-based relative scoring system that could be used during the follow-up of PNS wall erosion in patients with AFRS. Bone erosion is considered one of the minor criteria for a diagnosis of AFRS according to the Bent and Kuhn criteria; however, its presence is supportive of the diagnosis [1]. The incidence of bone erosion in patients with AFRS ranges from 20% to 56% in the literature [12,14,16-20].

In our study, 82 patients (57.7%) had bone erosion with expansion into at least one sinus wall. We investigated 142 patients, which was the same sample size as that in the study by Nussenbaum et al. [14]. The incidence of bone erosion in their study was 20% and most frequently involved the lamina papyracea. We studied each sinus individually and found that the one most commonly involved was the ethmoid sinus. The most frequent extra-sinus extension was to the orbit via the lamina papyracea, frontal floor, or maxillary roof erosion, followed by anterior cranial fossa extension due to an erosion of the roof of the ethmoid sinuses or the posterior table of the frontal sinuses. These findings are consistent with the results published by Nussenbaum et al. [14] and are further supported by White et al., who found that 52% of their sample had radiographic evidence of bony erosions, with the most frequently involved wall being the lamina papyracea [16].

In this study, we introduced a novel scoring system for AFRS-related bone erosion, i.e., the bone erosion score, which ranges from 0 to 72, for the clinically relevant PNS walls. Two rhinologists tested the consistency and validity of this scoring system by independently evaluating the CT images from 30 patients, with only three instances of disagreement. These disagreements were for grading in the region of the lamina papyracea at the anterior ethmoid sinus, the frontal sinus floor, and the lateral wall of the sphenoid sinus. Therefore, we consider BES as an objective tool for the assessment of disease severity, and it may assist in surgical planning, including the duration of surgery, patient counseling, and anesthesia considerations. Additionally, it may help assess the degree of bone regeneration, which will be evident from the results of an on-going follow-up study for evaluating the outcome of bone erosion and assessing the degree of bony regeneration over a period of time. Furthermore, we anticipate that the BES will be an important tool in future studies and facilitate communication between researchers and clinicians when assessing the severity of bone erosion and the degree of regeneration in patients with AFRS.

In the English literature, we found only one scoring system describing bone erosion in AFRS, which was proposed by Wise and colleagues [21]. In this system, the scores possible range from 0 to 24 according to the presence of bony erosions regardless of size. In contrast, our BES provides a more comprehensive description of bone erosion in patients with AFRS. The BES will be a valuable tool for determining the rate of bone regeneration and assessing its extent on a postoperative follow-up image.

## Conclusions

The orbit is the most frequent site of extra-sinus extension in AFRS and is usually caused by erosion of the lamina papyracea. We propose a novel image-based scoring system for AFRS-related bone erosion, i.e., the BES, which has a score range from 0 to 72 based on grading the erosion extension for each PNS wall from 1 to 3. We think this new scoring system will be an important tool in the assessment of disease severity and the longitudinal measurement of the degree of bone regeneration on CT post treatment in AFRS cases.
